# Translation and validation of the French version of the ObsQoR-10 questionnaire for the evaluation of recovery after delivery: the ObsQoR-10-French

**DOI:** 10.1016/j.bjao.2023.100221

**Published:** 2023-08-17

**Authors:** Éric Mazoué, Mathilde Veret, Romain Corroënne, Marie-Bénédicte Mercier, Henri Lomo, Caroline Verhaeghe, Sigismond Lasocki, Pierre-Emmanuel Bouet, Maxime Léger

**Affiliations:** 1 Department of Anaesthesia and Intensive Care, Angers University Hospital, France; 2 Department of Obstetrics and Gynaecology, Angers University Hospital, France; 3 Department of Anesthesia and Perioperative Care, UCSF, San Francisco, CA, USA

**Keywords:** Caesarean section, delivery, obstetrical anaesthesia, Patient Health Questionnaire, patient-reported outcome measures

## Abstract

**Background:**

The Obstetric Quality of Recovery-10 (ObsQoR-10) is a validated tool for assessing the quality of postpartum recovery. This study aimed to validate the French version of the ObsQoR-10 scale (ObsQoR-10-French).

**Methods:**

After translating the ObsQoR-10 into French, we conducted a psychometric validation involving internal consistency, convergent validity, construct validity, reliability, responsiveness, scaling properties, acceptability, and feasibility. French women who underwent either a vaginal delivery (spontaneous or induced labour), or an emergency or elective Caesarean section (C-section) were prospectively included. They completed the ObsQoR-10-French before delivery and at 24 h (H24) and 48 h (H48) after delivery.

**Results:**

Of the 500 women included, 431 (86%) completed the questionnaire at all three timepoints. A total of 352 women (82%) underwent vaginal delivery (with 228 [53%] experiencing spontaneous labour and 124 [29%] had labour induced), whereas 53 (12%) women underwent an emergency C-section and 26 (6%) an elective C-section. The ObsQoR-10-French demonstrated excellent internal consistency with a Cronbach's coefficient of 0.81, 95% confidence interval 0.78–0.84 at H24. The tool was correlated with an 11-item global health score (*P*<0.001). Of the list of hypotheses for evaluating the construct validity, 81% were confirmed (negative associations between ObsQoR-10-French and length of labour, hospital stay, the need for a C-section, and the emergency level of the C-section). The Cohen effect size at H24 was 0.58. The intra-class coefficient was 0.90, 95% confidence interval 0.86–0.93 at H24.

**Conclusion:**

The ObsQoR-10-French is a valid and reliable psychometric questionnaire, capable of assessing the quality of postpartum recovery in French-speaking populations.

**Clinical trial registration:**

NCT04489602.

The recovery process after delivery, whether through vaginal or Caesarean delivery, is a complex and multifaceted process that can lead to stress, anxiety, pain, and other major and minor complications, such as nausea or vomiting. Unfortunately, the traditional clinical criteria typically used to evaluate peripartum interventions focus only on a few morbidity variables, neglecting the overall recovery process.^1^^,^^2^ To address this gap, patient-centred self-report scales have been developed for post-surgical recovery, which evaluate recovery in a holistic manner. However, these scales do not consider the unique aspects of obstetrics and are therefore not suitable for evaluating postpartum recovery. Therefore, the use of criteria focused on overall recovery, particularly in the obstetric context, has become increasingly crucial.

In 2019, the English language Obstetric Quality of Recovery-11 (ObsQoR-11) scoring tool was developed in English and validated to assess the recovery of women who underwent elective or non-elective Caesarean section (C-section).^3^^,^^4^ To facilitate questionnaire completion, the tool was condensed into a 10-item version that merged the ‘severe pain’ and ‘moderate pain’ items. This ObsQoR-10 version was then validated for vaginal delivery and was found to be reliable, sensitive, unidimensional, and easily implemented in clinical practice.^5^^,^^6^ Various translations have already been validated.^7^, ^8^, ^9^, ^10^ Recently, the ObsQoR-10 questionnaire has been used in a nationwide study across 107 sites in the United Kingdom, aimed at evaluating postpartum recovery after peripartum anaesthesia interventions.^11^ French is the fifth most widely spoken language in the world, with ∼280 million regular users.^12^ The objective of our study was to validate the French version of the ObsQoR-10 questionnaire (ObsQoR-10-French) to measure the quality of early postpartum recovery in French-speaking populations.

## Methods

This study received approval from a French research ethics committee (Comité de Protection de Personnes Est-III, registration ID 2020-A02013-36), and the protocol was registered on ClinicalTrials.gov (NCT04489602). We conducted a monocentric prospective cohort study at Angers University Hospital in France from 3 February 2021 to 29 July 2021. Although written consent was waived, all patients were informed about the data collection as mandated by French law,^13^ and all agreed to its use. The methodology for questionnaire validation adhered to the COSMIN guidelines ‘Consensus-based standards for the selection of health measurement instruments’.^14^

### Study population

All participants had to meet specific inclusion criteria: being 18 yr or older, being a French speaker, and consenting to the use of their data. They were required to understand and complete the ObsQoR-10-French questionnaire at inclusion, either independently or with the help of an assessor. They needed to be admitted to the labour room for an elective C-section, spontaneous or induced labour. Patients with significant psychiatric or neurological disorders that might compromise their cooperation in completing the questionnaire, or women with psychological risks related to a specific obstetrical context, such as *in utero* fetal death or medical abortion, were not included. We identified eligible women at labour room admission. Patient enrolment was undertaken either in the induction room (before an elective C-section) or after the achievement of epidural analgesia for the other women. The convenience sample was recruited when the investigators were available.

### Development and pilot testing of the French Obstetrical Quality of Recovery-10: ObsQoR-10-French

After obtaining authorisation from S. Ciechanowicz and P. Sultan (via e-mail, 5 May 2020), the authors of the ObsQoR-11 and the revised ObsQoR-10 versions, the ObsQoR-10 questionnaire was independently translated into French by two investigators fluent in both French and English, following existing recommendations.^15^ We compared and combined the two translated questionnaires to create a pilot version of the questionnaire in French. This pilot version was then back translated into English by two independent bilingual translators to verify the consistency of the translated items (forward translation/backward translation method). The pilot questionnaire and the backward translation were reviewed by the four translators to finalise the translated questionnaire. Approximately 20 patients and 10 health professionals (midwives and care assistants) with experience in immediate postpartum care completed the translated version to evaluate comprehension of the items. During this phase, we assessed the content validity (i.e. the degree to which the instrument adequately reflects the construct) through qualitative interviews (with EM or ML). To clarify the questionnaire and optimise completion, we included the emojis added for the validation of the ObsQoR-11 questionnaire for non-elective C-sections.^4^ This phase enabled us to develop the ObsQoR-10-French version tested in the current study (Supplementary material). The questionnaire's subscales are physical comfort (*n*=3 items), emotional state (*n*=2 items), pain (*n*=1 item), physical independence (*n*=2 items), and the ability to take care of the newborn (*n*=2 items). Each item is scored on a scale of 0–10, except for the first four items, which are graduated from 10 to 0. The total score is the sum of all item scores (i.e. a score ranging from 0 to 100, where 0 is the worst recovery score, and 100 indicates the best).

### Available data

Women completed the ObsQoR-10-French questionnaire on paper before delivery (baseline status=H0), and at 24 (H24) and 48 (H48) h after delivery. The questionnaire was filled out at H0 during labour after the initiation of epidural analgesia (if any) or just before the C-section (in the case of a scheduled C-section). They completed the questionnaire independently, otherwise with the help of an assessor. If the patient had returned home, an assessor interviewed her by phone. Assessments at H24 and H48 were our measures of interest. These assessments were typically conducted within a range of plus or minus 4 h and were generally avoided during the middle of the night. At baseline, we collected patient characteristics such as weight, height, age, American Society of Anesthesiologists (ASA) physical status (PS), co-morbidities, smoking status, alcohol or any other history of a substance use disorder, the number of pregnancies, number and type of previous deliveries, obstetric comorbidities or outcomes (malformation, pre-eclampsia, *in utero* growth retardation), use of assisted reproductive technology, and participation in specific childbirth education classes. We recorded the duration of labour, type of delivery, emergency level in case of non-elective C-section, the need for operative vaginal delivery, the type of anaesthesia, the occurrence of delivery complications (haemorrhage, eclampsia, amniotic embolism), the number of newborns, and their need for intensive care. At H24 and H48, we noted the postpartum-specific complications (postpartum psychological disorder, thromboembolic, and breastfeeding complications) and complications according to the PostOperative Morbidity Survey (POMS) classification.^16^ Each woman was also asked to rate her global health at each interval (H0, H24, and H48), measured by a verbal analogue scale (VAS) ranging from 0 (very poor global condition) to 10 (best global condition). We timed the duration of questionnaire completion at H24. At H24, we asked patients to complete the ObsQoR-10-French twice at intervals of 30–60 min to assess test-retest reliability. At H24 and H48, patients had to rate their recovery over the last 24 h on a seven-item Likert scale ranging from ‘very poor’ to ‘very good’. We used the difference in the mean ObsQoR-10-French between ‘same’ and ‘slightly good’ to determine the minimum clinically important difference (MCID) using to the anchor-based method.^17^ Women also had to answer the question ‘Do you consider that you have recovered well?’ (yes/no response) at H24 and H48. We used these data to determine the clinically significant differences (CSDs).^18^

### The psychometric validation

We conducted a comprehensive psychometric evaluation to validate the ObsQoR-10-French questionnaire.^14^^,^^19^

- **Content validity** refers to the extent to which the questionnaire items effectively represent the concept. The target population for the ObsQoR-10-French includes all women admitted for labour or elective C-section.
- **Internal consistency** reflects the extent to which the items measure an underlying construct.
- **Structural validity** is the degree to which the scores from the instrument adequately represent the dimensionality of the construct to be measured. We presumed the unidimensionality of the ObsQoR-10-French questionnaire.
- **Criterion validity** is the association between the measurement questionnaire and a ‘gold standard’ scale. In the absence of the latter, we tested convergent validity by comparing the ObsQoR-10-French, at H24 and H48, with a global health visual analogue scale (VAS), which ranges from 0 (very poor global condition) to 10 (best global condition).
- **Construct validity** refers to the consistency of scores in relation to the theoretical predictions of changes associated with the concept. For this purpose, we tested several hypotheses, and >75% of our assumptions had to be confirmed. We assumed that ObsQoR-10-French scores at H24 and H48 were inversely associated with labour duration and length of hospital stay. Women who delivered by non-elective C-section would have lower scores than those who delivered by elective ones. Women with complications would have a lower ObsQoR-10-French score and those with restricted contact with their child in the early period (hospitalisation in intensive care unit or neonatology). The score value would not vary with age. We assumed that the score value was positively associated with the number of prior deliveries.
- **Reliability** entails repeat testing on stable individuals providing similar answers. The agreement concerns the absolute measurement error, whereas reliability is the degree to which patients can be distinguished from each other, despite measurement error.
- **Responsiveness** denotes the capacity of a questionnaire to identify clinically significant changes over time. Our goal was to gauge the effectiveness of the questionnaire in assessing the repercussions of delivery. Certain questions in the ObsQoR-10-French are specific to the postpartum period, rendering them unsuitable for the prepartum period (e.g. holding and feeding the baby). To establish a baseline state before delivery, we requested women to envisage their ability to hold/feed their baby at that time.
- We detected **floor or ceiling effects** if >15% of respondents scored the lowest or highest possible score.^20^
- **Acceptability and feasibility** represent measures of user-friendliness, including the patient recruitment rate, the total participation rate within the three time frames (H0, H24, and H48), and the duration taken to complete the questionnaire (stopwatch by research assistant).

### Sample size calculation

We established the sample size at 500 patients for the French validation of ObsQoR-10, aiming to include a subgroup of nearly 100 women who had given birth by C-section (the minimum sample size in other translations of the ObsQoR-10 questionnaire for this subgroup). We assumed this would account for 20% of the total population. Furthermore, the threshold of 300 patients appeared to be an acceptable limit for studying the dimensions of a measurement scale.^21^ The planned enrolment of 500 patients would allow us to exceed the minimal threshold of 300, even with 10% loss of subjects or missing data.

### Statistical analysis

We present data as mean (standard deviation) (sd) or median [inter-quartile range] for quantitative variables. Qualitative variables are represented by the number of women and the percentage (%). For comparisons of categorical variables, χ^2^ tests were used (if numbers were inadequate, Fisher's exact tests were preferred). For comparisons of continuous variables, we used the Student *t*-test for variables following a normal distribution; otherwise, we used the Wilcoxon test.

We proposed an inter-item correlation matrix composed of Pearson correlation coefficients. Associations between quantitative variables were measured using Pearson correlation coefficients. We explored the number of dimensions of the questionnaire by the total percentage variance explained by the first factor. To maintain the hypothesis of the unidimensionality of the scale, our criterion was a total percentage variance exceeding 25%. Internal consistency was assessed using Cronbach's ⍺.^22^ The aim was to achieve a value between 0.70 and 0.90.^23^ Test–retest reliability was evaluated using the agreement intraclass correlation coefficient (agreement ICC).^24^ A value of 0.70 is generally recommended as a minimum standard for reliability.^23^ The measurement error is expressed as the standard error of measurement (SEM), which includes systematic differences (i.e. SEM agreement).^25^ Responsiveness was quantified using the Cohen effect size (average change score divided by the sd at baseline),^26^ and standardised response mean (change scores divided by the sd of the change scores).^27^ The final MCID was the average of the MCID obtained by the distribution methods (corresponding to 1.96×SEM and representing a larger variation than the random variation at 5% of uncertainty) and anchoring methods (the difference in mean score values between the ‘same’ and ‘slightly good’ statuses). The CSD was the difference in ObsQoR-10-French score between the group of women who answered ‘Yes’ to the question ‘Do you consider that you have recovered well?’ and those who answered ‘No’.

The 95% confidence intervals (95% CIs) were determined by bootstrapping. We rejected the null hypothesis if the *P*-value was <0.05. No procedure for correcting for multiple statistical tests was implemented, as the analyses were primarily for exploratory purposes. We performed all statistical analyses using R software (version 3.6.3, R Foundation for Statistical Computing, Vienna, Austria).

## Results

### Description of the population

**Fig 1:**
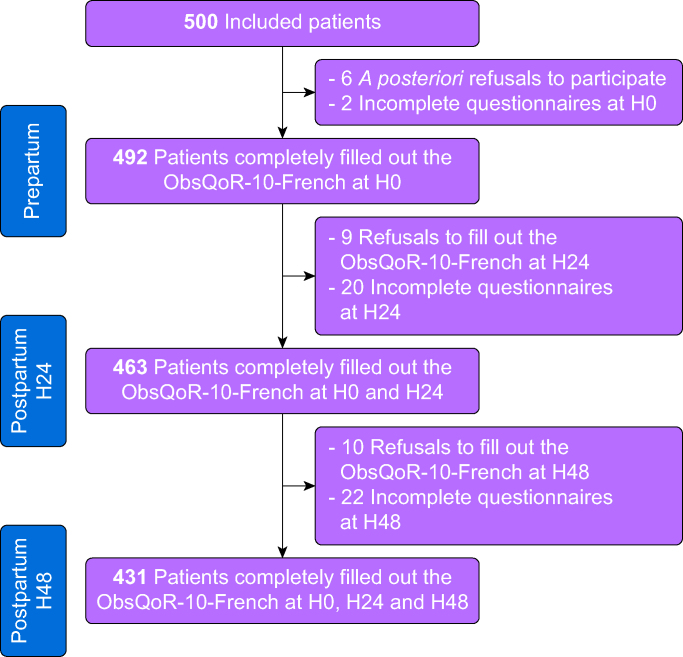
Flow chart. ObsQoR-10-French, Obstetric Quality of Recovery-10-French.

During the study period, 500 patients were included for the ObsQoR-10-French validation. Our sample was one of convenience, with a total of 1870 women having given birth during the specified period. Finally, 431 (86.2%) patients completed the questionnaire at H0, H24, and H48. The flow chart is depicted in Figure 1. Patient characteristics are summarised in Table 1, with additional details in Supplementary Tables S1 and S2. Supplementary Table S3 compares the main characteristics between the analysed (431 women, 86.2%) and non-analysed women (69 women, 13.8%). The non-analysed group had fewer childbirth education classes, longer labour, and longer hospital stay. The average patient age was 30.2 (5.2) yr, with a predominance of ASA-PS 2 patients (397 patients, 92.1%). A total of 352 women (82%) had vaginal deliveries (including 228 [53%] spontaneous labours and 124 [29%] induced labours), 53 (12%) women underwent emergency C-sections (either after spontaneous or induced labour), and 26 (6%) elective C-sections.

**Table 1:** Characteristics of the patients. ASA-PS, American Society of Anesthesiologists physical status; BMI, body mass index; CS, Caesarean section; ObsQoR-10-French, Obstetric Quality of Recovery-10-French; VD, vaginal delivery.

	Overall *n*=431	Spontaneous VD *n*=228	Induced VD *n*=124	Elective CS *n*=26	Non-elective CS *n*=53	*P*-value
Medical history						
Age (yr)	30.0 [26.0–34.0]	30.0 [26.0–33.0]	30.0 [26.0–34.0]	33.0 [28.0–37.0]	31.0 [28.0–34.0]	0.031
BMI (kg m^−2^)	25.8 (6.1)	26.7 (6.2)	24.7 (5.4)	26.6 (7.5)	28.0 (7.2)	0.001
ASA-PS score						
ASA-PS 2	397 (92.1)	215 (94.3)	112 (90.3)	24 (92.3)	48 (90.6)	0.574
ASA-PS 3	34 (7.9)	13 (5.7)	12 (9.7)	2 (7.7)	5 (9.4)	
Cardiovascular history	3 (0.7)	3 (1.3)	0 (0.0)	0 (0.0)	0 (0.0)	0.442
Respiratory history	20 (4.6)	10 (4.4)	8 (6.5)	1 (3.8)	1 (1.9)	0.594
Diabetes	23 (5.3)	5 (2.2)	8 (6.5)	1 (3.8)	9 (17.0)	<0.001
Tobacco consumption	32 (7.4)	17 (7.5)	8 (6.5)	1 (3.8)	6 (11.3)	0.609
Tobacco consumption during pregnancy	20 (4.6)	11 (4.8)	7 (5.6)	0 (0.0)	2 (3.8)	0.647
Substance use disorder	2 (0.5)	1 (0.4)	0 (0.0)	0 (0.0)	1 (1.9)	0.388
Obstetric history						
Number of pregnancies						<0.001
1	146 (33.9)	35 (28.2)	88 (38.6)	4 (15.4)	19 (35.8)	
2	122 (28.3)	31 (25.0)	66 (28.9)	7 (26.9)	18 (34.0)	
3	83 (19.3)	26 (21.0)	41 (18.0)	8 (30.8)	8 (15.1)	
4	40 (9.3)	14 (11.3)	20 (8.8)	2 (7.7)	3 (5.7)	
>4	40 (9.3)	18 (14.5)	13 (5.7)	5 (19.2)	5 (9.4)	
Fetal malformation	6 (1.4)	3 (1.3)	1 (0.8)	2 (7.7)	0 (0.0)	0.035
Preeclampsia	9 (2.1)	1 (0.4)	4 (3.2)	1 (3.8)	3 (5.7)	0.057
Intrauterine growth restriction	15 (3.5)	2 (0.9)	9 (7.3)	3 (11.5)	1 (1.9)	0.002
Assisted reproductive technologies	11 (2.6)	5 (2.2)	1 (0.8)	2 (7.7)	3 (5.7)	0.091
Childbirth education class	194 (45.0)	107 (46.9)	61 (49.2)	5 (19.2)	21 (39.6)	0.133
Operative vaginal delivery	83 (19.3)	41 (18.0)	25 (20.2)	—	5 (9.4)	0.220
Length of labour (h)	11.2 (7.4)	9.0 (5.6)	10.3 (5.3)	—	12.5 (7.9)	<0.001
Length of stay (days)	4 [3–4]	3 [3–4]	4 [3–4]	6 [5–9]	5 [4–6]	<0.001
Delivery complications						
Postpartum haemorrhage	61 (14.2)	19 (8.3)	20 (16.1)	6 (23.1)	16 (30.2)	<0.001
Drug allergy	1 (0.2)	0 (0.0)	1 (0.8)	0 (0.0)	0 (0.0)	0.479
Eclampsia	2 (0.5)	0 (0.0)	2 (1.6)	0 (0.0)	0 (0.0)	0.174
Amniotic embolism	1 (0.2)	1 (0.4)	0 (0.0)	0 (0.0)	0 (0.0)	0.827
ObsQoR-10-French						
Prepartum score	85.5 (0.8)	84.1 (11.4)	88.0 (10.0)	89.9 (8.0)	83.6 (10.4)	0.001
H24 score	79.2 (5.0)	82.1 (12.1)	80.9 (14.8)	74.9 (14.7)	65.0 (18.4)	<0.001
H48 score	89.4 (11.3)	91.3 (8.8)	89.8 (11.6)	87.4 (12.8)	80.1 (14.8)	<0.001

### Internal consistency and structural validity

**Fig 2:**
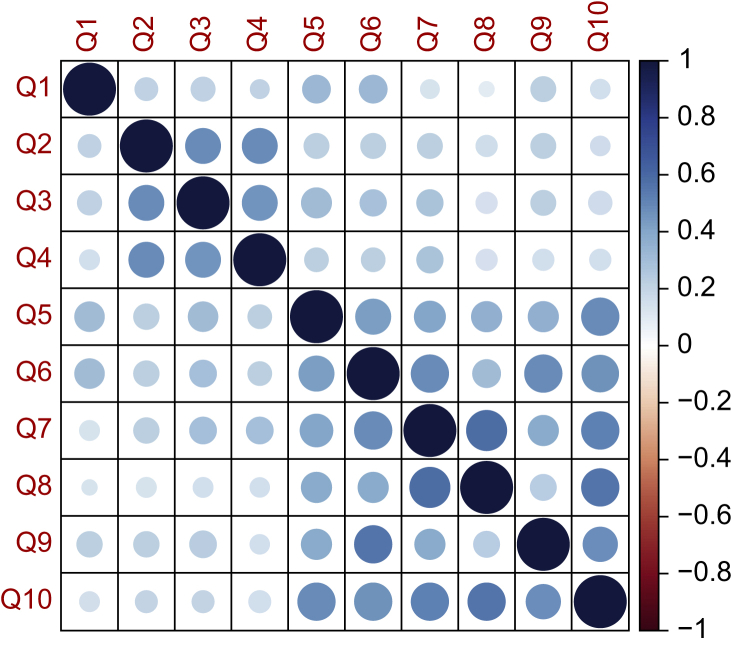
Heatmap of inter-item correlations for the ObsQoR-10-French score at 24 h after delivery. The 10 questions composing the ObsQoR-10-French questionnaire (from Q1 to Q10) are distributed on the axes. The strength of the correlation between the items is represented by the circle size (larger circle for stronger association) and by the colour shade. Negative correlations are represented in red, and positive correlations in blue. ObsQoR-10-French, Obstetric Quality of Recovery-10-French.

The heatmap representation of the inter-item correlations of ObsQoR-10-French at H24 is depicted in Figure 2. That for the ObsQoR-10-French at H48 is reported in Supplementary Figure S1. Supplementary Tables S4 and S5 present the inter-item correlation tables. The overall average inter-item correlation was 0.30 with 95% CI 0.27–0.30 at H24 and 0.33 with 95% CI 0.29–0.37 at H48. The exploratory analysis confirmed the unidimensionality of the ObsQoR-10-French, both at H24 (39.8% variance for the first dimension) and H48 (46.9% variance for the first dimension). The scree plot representations are depicted in Supplementary Figures S2 and S3. Cronbach's coefficients were 0.81 with 95% CI 0.78–0.84 at H24 and 0.83 with 95% CI 0.79–0.86] at H48.

### Convergent validity

The ObsQoR-10-French and the global health scores were correlated with coefficients of 0.45 with 95% CI 0.37–0.52 at H0, 0.59 with 95% CI 0.53–0.65 at H24, and 0.69 with 95% CI 0.63–0.73 at H48.

### Construct validity

Overall, of the eight hypotheses of association with the ObsQoR-10-French score assessed at 24 and 48 h after delivery, 81% were confirmed. The results of the assumptions tested on the ObsQoR-10-French are reported in Table 2.

**Table 2:** The summary table of tested assumptions (constructed validity) on the global ObsQoR-10-French score at 24 and 48 h after delivery. Tests of quantitative variables based on Pearson's correlation coefficient. Tests of binary qualitative variables based on Student's *t*-test. Values are Pearson's correlation coefficients with [95% confidence interval] or means (standard deviations). C-section, Caesarean section; ObsQoR-10-French, Obstetric Quality of Recovery-10-French.

Assumptions	ObsQoR-10-French at 24 h	Confirmed	ObsQoR-10-French at 48 h	Confirmed
No association between the age and ObsQoR-10-French	−0.040 [−0.134 to −0.054], *P*=0.398	X	−0.080 [−0.173 to 0.014], *P*=0.096	X
Positive association between the number of prior pregnancies and ObSQoR-10-French	0.124 [0.030–0.216], *P*=0.010	X	0.173 [−0.029 to 0.159], *P*=0.173	
Negative association between length of labour and ObsQoR-10-French	−0.198 [−0.286 to −0.105], *P*<0.0001	X	−0.203 [−0.292 to −0.111], *P*<0.0001	X
Negative association of the delivery mode on ObsQoR-10-French (vaginal delivery *vs* C-section)	81.7 (13.1) *vs* 68.0 (17.8), *P*<0.001	X	90.9 (9.5) *vs* 83.0 (14.2), *P*<0.001	X
Negative association between the need of emergency C-section and ObsQoR-10-French (elective C-section *vs* scheduled C-section)	73.5 (14.4) *vs* 65.6 (18.7), *P*=0.046	X	87.3 (11.8) *vs* 81.1 (14.8), *P*=0.052	
Negative association between the occurrence of postpartum complications and ObsQoR-10-French	79.4 (14.9) *vs* 73.4 (19.9), *P*=0.399		89.8 (10.6) *vs* 78.1 (15.1), *P*=0.016	X
Negative association between the need of neonatal hospital unit and ObsQoR-10-French (no need *vs* neonatal hospital unit)	79.7 (14.7) *vs* 64.8 (17.5), *P*<0.013	X	89.8 (10.5) *vs* 75.5 (17.4), *P*<0.013	X
Negative association between the hospital length of stay and ObsQoR-10-French	−0.177 [−0.296 to −0.081], *P*<0.001	X	−0.238 [−0.328 to −0.144] *P*<0.001	X

### Reliability

By comparing the questionnaires completed with 30 min to 1 h time difference, we calculated an agreement ICC of 0.90 with 95% CI 0.86–0.93. The SEM was 4.5 with 95% CI 4.1–4.8.

### Responsiveness

**Fig 3:**
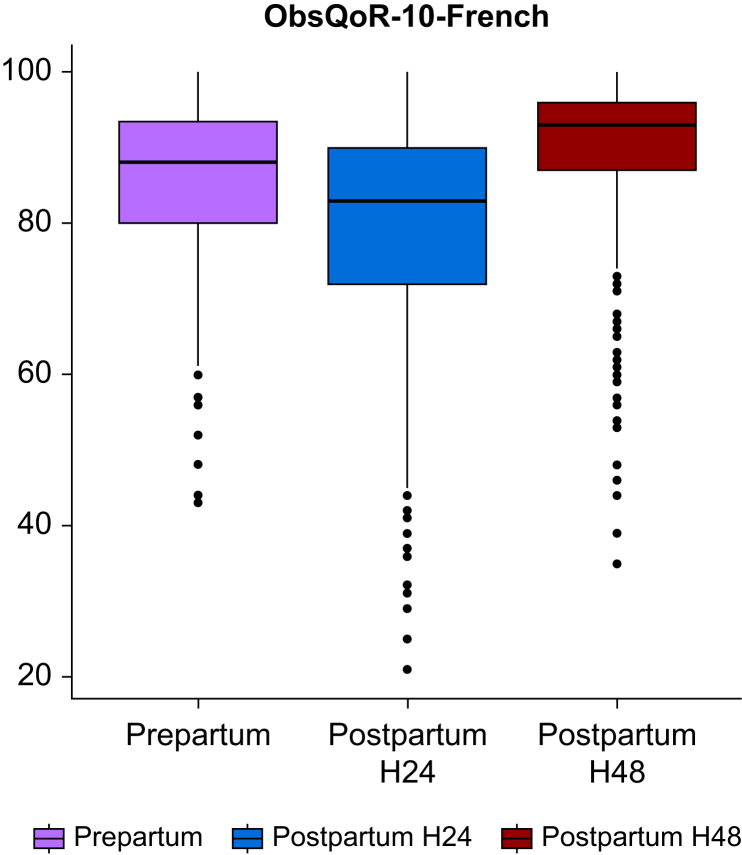
Distribution of the ObsQoR-10-French at three times: prepartum, 24 h and 48 h after delivery. Each box displays the inter-quartile range (IQR), from the first quartile (Q1, 25th percentile) to the third quartile (Q3, 75th percentile), with the median (Q2, 50th percentile) marked as a line within the box. The ‘whiskers’ extend to the smallest and largest data points not considered outliers, which are individually marked beyond the whiskers. Outliers (points) fall below Q1−1.5*IQR or above Q3+1.5*IQR. ObsQoR-10-French, Obstetric Quality of Recovery-10-French.

Before delivery, the average ObsQoR-10-French score was 85.5 (10.8), *vs* 79.2 (15.0) at H24, and 89.4 (11.3) at H48. The distribution of the global ObsQoR-10-French score at these three points in time is displayed in Figure 3. This distribution by mode of delivery is also represented in Supplementary Figure S4. At H24, Cohen's effect size for the ObsQoR-10-French was 0.58, with a standardised response mean of 0.33. All items decreased in value between baseline and H24. Table 3 summarises the responsiveness between baseline and H24. Responsiveness between baseline and H48 is presented in Supplementary Table S6.

**Table 3:** Items responsiveness of the ObsQoR-10-French between prepartum phase and 24 h after delivery. ∗Values are means (standard deviations). ^†^Mean changes are represented with [95% confidence interval]. ObsQoR-10-French, Obstetric Quality of Recovery-10-French.

ObsQoR-10-French items	Mean values before delivery∗	Mean values at H24∗	Mean changes in values^†^	% Change from baseline	Cohen effect size	Standardised response mean
Q1	5.8 (2.8)	5.5 (2.4)	0.28 [−0.09 to 0.61]	5.2	0.1	0.07
Q2	9.1 (2.1)	8.9 (2.2)	0.22 [−0.06 to 0.5]	2.2	0.11	0.08
Q3	9.7 (1)	8.8 (2)	0.91 [0.69–1.12]	9.3	0.88	0.40
Q4	9.2 (2)	8.9 (2.3)	0.27 [−0.01 to 0.52]	3.3	0.14	0.09
Q5	7.2 (2.3)	6.7 (2.1)	0.47 [0.20–0.80]	6.9	0.2	0.15
Q6	9.2 (1.6)	7.9 (2.7)	1.37 [1.09–1.69]	14.1	0.87	0.44
Q7	8.6 (2.4)	8.5 (2.6)	0.07 [−0.26 to 0.41]	1.2	0.03	0.02
Q8	8.7 (2.4)	7.8 (2.9)	0.87 [0.53–1.23]	10.3	0.36	0.22
Q9	9.7 (1.2)	8.6 (2.8)	1.06 [0.79–1.35]	11.3	0.86	0.35
Q10	8.4 (2.1)	7.7 (2.3)	0.72 [0.42–1.02]	8.3	0.34	0.24
Total	85.6 (10.8)	79.3 (15)	6.24 [4.56–7.98]	7.4	0.58	0.33

### Scaling properties

No major ceiling or floor effect was observed. Histograms showing the distribution of ObsQoR-10-French scores at H24 and H48 are provided in Supplementary Figures S5 and S6.

### Acceptability and feasibility

Among the included patients, 99% of women agreed to participate and completed the baseline questionnaire in its entirety. Ninety-four percent completed both the baseline and H24 ObsQoR-10-French scale, and 87% filled out the questionnaire at all three time points (Fig. 1). The average time to fully complete the questionnaire was 3 (3) min.

### Clinical differences

The MCID at H24 and H48 were 5.0 (95% CI 2.3–7.8) and 4.5 (95% CI 1.1–8.0), respectively. The CSD at H24 and H48 were 18.2 (95% CI 16.9–19.5) and 20.3 (95% CI 19.40–21.18), respectively.

## Discussion

This study entailed the development and prospective validation of a French translation of the ObsQoR-10, a 10-item questionnaire that is used to assess postpartum recovery. The ObsQoR-10-French, crafted through translation and cross-cultural adaptation, is unidimensional, valid, sensitive, reproductible, and demonstrates robust acceptability, feasibility, and scaling properties.

Our cohort comprised a heterogeneous population, reflecting both spontaneous and induced labour types and various delivery methods (vaginal, scheduled, and emergency C-section). This diversity allowed us to highlight the excellent psychometric properties of ObsQoR-10-French. The ObsQoR-10-French score at 24 and 48 h after delivery was lower in induced than in spontaneous vaginal delivery and was even lower for C-section. Thus, women exhibited better early recovery in vaginal delivery, with a mean value of 82.1, aligning with the findings from the original studies of the ObsQoR-10 questionnaire.^5^^,^^6^ Analysing each score item in our population revealed that pain and comfort limitation majorly contributed to recovery impairment, mirroring the findings in the British and US populations.^5^^,^^6^

All psychometric validities were reaffirmed, similar to other translations of the ObsQoR-10 questionnaire. For the ObsQoR-10-French convergent validity assessment, we observed only a moderate correlation with the global health VAS. This result aligns with the original ObsQoR-10 validations where correlations were moderate.^5^^,^^6^ Similar findings were also noted in other translations. This moderate association strength between ObsQoR-10 values and global health underscores the uniqueness of postpartum recovery, inadequately captured by non-specific scales. A specific patient-centred measure should evaluate postpartum recovery to address concepts of comfort, self-control, and even the ability to feed and hold the baby. The score increased between H24 and H48, reflecting the dynamics of recovery and confirming responsiveness after delivery or C-section. Our responsiveness assessment revealed a better ObsQoR-10-French average result at H48 (i.e. a mean value of 89.4) than at H0 (i.e. a mean value of 85.5). We attribute this difference to the majority of H0 assessments being performed during labour. Even though we waited for epidural anaesthesia, the stressful and painful context may have contributed to a lower score. Therefore, our baseline assessment at H0 did not represent a ‘normal’ state and was already influenced by the labour. Unfortunately, we cannot fully confirm this assumption as the initial studies validating the ObsQoR-10 score in vaginal deliveries did not perform this baseline measurement.^5^^,^^6^ Moreover, recent translations into other languages have not validated the vaginal delivery modality.

Another strength of our study was the assessment of the MCID and CSD, which could be used to design future randomised trials assuming a minimal or significant clinical effect for evaluating a therapeutic intervention. Nevertheless, these values are specific to this population and may not be universally applicable.

As noted in the methods section, we inverted the score for the four first items (e.g. 10 for a low pain score and 0 for a high one). This reversal confused several patients accustomed to rating their pain differently (i.e. 10 for a high pain score) and necessitated the investigator's assistance. Therefore, we suggest not making this inversion in future applications or using a computerised version to calculate the global score without the risk of error.

Although we focused solely on the first 48 h to validate this translation in the context of assessing early postpartum recovery, it would be interesting to determine whether a low score correlates with an unfavourable long-term evolution. Similar findings were observed in other patient-reported outcome measures, such as QoR-15, where a low score was associated with postoperative complications after the procedure.^28^ Other patient-related outcome measures specific to obstetrics might be more appropriate for assessing longer-term recovery.^29^ Nonetheless, the ObsQoR-11 questionnaire is already applied as a study outcome,^30^ and its use will probably expand, as was the case for the QoR-15 or QoR-40 for surgery. Beyond this purpose, the ObsQoR-10 could be applied more systematically to monitor the recovery as part of continuous practice improvement or for audits. In addition, some authors recommend its use to evaluate the enhanced recovery after C-sections.^31^ The ObsQoR-10 questionnaire has been included in a recently published core outcome set of metrics that should be used to evaluate the impact of enhanced recovery protocols in future research studies.^32^

Our study also has some limitations. Firstly, our single-centre validation does not assure the generalisation of our translated version. Secondly, 69 (13.8%) patients did not complete the questionnaire at the three time points. However, the 431 (86.2%) final participants are ample to confirm good acceptability. Even so, we did not reach our inclusion goal of 100 patients who had a C-section, which may limit the questionnaire's validation in this subgroup. Thirdly, enrolments could be performed 24 h a day, every day. With a reduced staff team at night and on the weekend, fewer inclusions might have happened during these periods. Although this strengthened the population's heterogeneity and generalisability, there might have been an inclusion bias. In addition, because the anaesthesia team performed the enrolment, the majority of patients were included after establishing epidural analgesia. Consequently, the proportion of vaginal delivery without analgesia or anaesthesia is low, creating another selection bias. Fourthly, our study is not without further methodological limitations: we opted to use an 11-item global health scale instead of the validated 100-point scale recommended by EuroQoL, the estimation of the CSD was done through a non-validated question, and we did not perform cross-cultural validity using differential item functioning. Fifthly, including data on episiotomies could have offered additional insight into their influence on the postpartum recovery. Finally, French is spoken in other countries with different cultural aspects that might influence the questionnaire's psychometric validity. It would be interesting to evaluate ObsQoR-10-French in those specific contexts.

## Conclusions

Our study validated the ObsQoR-10-French questionnaire for assessing postpartum recovery. This scale demonstrated excellent psychometric characteristics. We recommend its use in the French-speaking population for future clinical studies or to optimise the recovery process after delivery.

## Authors’ contributions

Patient recruitment: EM, HL

Data collection: EM, MV, MBM, HL

Analysis, writing—original draft: EM

Study design, questionnaire translation: RC, CV

Revision of draft of the paper, supervision: SL, PEB

Conceptualisation, methodology, analysis, and visualisation writing—original draft, supervision, project administration: ML

## Declarations of interest

The authors declare that they have no conflicts of interest.
