# Supplementary material for: Translation and validation of the French version of the ObsQoR-10 questionnaire for the evaluation of recovery after delivery: the ObsQoR-10-French
Source: BJA Open. 2023 Aug 17;7:100221. doi: 10.1016/j.bjao.2023.100221 (PMC10457490; doi:10.1016/j.bjao.2023.100221)

**Table S1.** Obstetrical and anaesthetic characteristics

|  | **Overall**  **(n = 431)** | **Induced VD**  **(n = 124)** | **Spontaneous VD**  **(n = 228)** | **Elective CS**  **(n = 26)** | **Non-elective CS**  **(n = 53)** | **p** |
| --- | --- | --- | --- | --- | --- | --- |
| **Obstetrical history** |  |  |  |  |  |  |
| C-Section emergency level |  |  |  |  |  |  |
| < 15min | - | - | - | - | 6 (11.3) |  |
| <30 min | - | - | - | - | 27 (50.9) |  |
| < 1h | - | - | - | - | 20 (37.7) |  |
| **Anaesthesia** |  |  |  |  |  |  |
| No anaesthesia | 1 (0.2) | 0 (0.0) | 1 (0.4) | 0 (0.0) | 0 (0.0) | 0.827 |
| Epidural anaesthesia | 404 (93.7) | 124 (100.0) | 226 (99.1) | 3 (11.5) | 51 (96.2) | <0.001 |
| Spinal anaesthesia | 26 (6.0) | 1 (0.8) | 0 (0.0) | 22 (84.6) | 3 (5.7) | <0.001 |
| General anaesthesia | 0 (0) | 0 (0) | 0 (0) | 0 (0) | 0 (0) | - |
| **New-born data** |  |  |  |  |  |  |
| Weight at birth (g) | 3262 ± 499 | 3257 ± 524 | 3275 ± 433 | 3058 ± 685 | 3314 ± 592 | 0.169 |
| Apgar score |  |  |  |  |  | 0.425 |
| 10 | 418 (97.0) | 122 (98.4) | 221 (96.9) | 26 (100.0) | 49 (92.5) |  |
| 9 | 6 (1.4) | 2 (1.6) | 3 (1.3) | 0 (0.0) | 1 (1.9) |  |
| 8 | 4 (0.9) | 0 (0.0) | 2 (0.9) | 0 (0.0) | 2 (3.8) |  |
| 6 | 1 (0.2) | 0 (0.0) | 1 (0.4) | 0 (0.0) | 0 (0.0) |  |
| 4 | 1 (0.2) | 0 (0.0 | 0 (0.0) | 0 (0.0) | 1 (1.9) |  |
| 1 | 1 (0.2) | 0 (0.0) | 1 (0.4) | 0 (0.0) | 0 (0.0) |  |
| Twins | 9 (2.1) | 5 (4.0) | 1 (0.4) | 1 (3.8) | 3 (5.7) | 0.045 |
| Neonatal hospital unit at H24 | 12 (2.8) | 0 (0.0) | 5 (2.2) | 4 (15.4) | 3 (5.7) | <0.001 |
| Neonatal hospital unit at H48 (%) | 12 (2.8) | 1 (0.8) | 5 (2.2) | 4 (15.4) | 2 (3.8) | 0.001 |

*CS, Caesarean Section; VD, Vaginal Delivery*

**Table S2.** Postpartum complications at 24 and 48 hours.

|  | **Overall**  **(n = 431)** | **Induced VD**  **(n = 124)** | **Spontaneous VD**  **(n = 228)** | **Elective CS**  **(n = 26)** | **Non-elective CS**  **(n = 53)** | **p** |
| --- | --- | --- | --- | --- | --- | --- |
| **POMS complications at H24** |  |  |  |  |  |  |
| Pulmonary | 1 (0.2) | 0 (0.0) | 1 (0.4) | 0 (0.0) | 0 (0.0) | 0.827 |
| Infectious | 0 (0.0) | 0 (0.0) | 0 (0.0) | 0 (0.0) | 0 (0.0) | - |
| Renal | 1 (0.2) | 0 (0.0) | 0 (0.0) | 1 (3.8) | 0 (0.0) | 0.001 |
| Digestive | 1 (0.2) | 0 (0.0) | 0 (0.0) | 0 (0.0) | 1 (1.9) | 0.067 |
| Cardio-vascular | 0 (0.0) | 0 (0.0) | 0 (0.0) | 0 (0.0) | 0 (0.0) | - |
| Neurologic | 0 (0.0) | 0 (0.0) | 0 (0.0) | 0 (0.0) | 0 (0.0) | - |
| Hematologic | 0 (0.0) | 0 (0.0) | 0 (0.0) | 0 (0.0) | 0 (0.0) | - |
| Operative site complication | 0 (0.0) | 0 (0.0) | 0 (0.0) | 0 (0.0) | 0 (0.0) | - |
| Pain requiring opioids or LRA^‡^ | 1 (0.2) | 0 (0.0) | 0 (0.0) | 1 (3.8) | 0 (0.0) | 0.001 |
| **Other complications at H24** |  |  |  |  |  |  |
| Psychiatric | 3 (0.7) | 2 (1.6) | 1 (0.4) | 0 (0.0) | 0 (0.0) | 0.516 |
| Breastfeeding | 3 (0.7) | 2 (1.6) | 0 (0.0) | 0 (0.0) | 1 (1.9) | 0.224 |
| Thrombo-embolic | 0 (0.0) | 0 (0.0) | 0 (0.0) | 0 (0.0) | 0 (0.0) | - |
| **POMS complications at H48** |  |  |  |  |  |  |
| Pulmonary | 1 (0.2) | 0 (0.0) | 1 (0.4) | 0 (0.0) | 0 (0.0) | 0.827 |
| Infectious | 3 (0.7) | 2 (1.6) | 1 (0.4) | 0 (0.0) | 0 (0.0) | 0.516 |
| Renal | 1 (0.2) | 0 (0.0) | 0 (0.0) | 1 (3.8) | 0 (0.0) | 0.001 |
| Digestive | 1 (0.2) | 0 (0.0) | 0 (0.0) | 0 (0.0) | 1 (1.9) | 0.067 |
| Cardio-vascular | 0 (0.0) | 0 (0.0) | 0 (0.0) | 0 (0.0) | 0 (0.0) | - |
| Neurologic | 0 (0.0) | 0 (0.0) | 0 (0.0) | 0 (0.0) | 0 (0.0) | - |
| Hematologic | 0 (0.0) | 0 (0.0) | 0 (0.0) | 0 (0.0) | 0 (0.0) | - |
| Operative site complication | 0 (0.0) | 0 (0.0) | 0 (0.0) | 0 (0.0) | 0 (0.0) | - |
| Pain requiring opioids or LRA^‡^ | 1 (0.2) | 0 (0.0) | 0 (0.0) | 1 (3.8) | 0 (0.0) | 0.001 |
| **Other complications at H48** |  |  |  |  |  |  |
| Psychiatric | 6 (1.4) | 2 (1.6) | 2 (0.9) | 0 (0.0) | 2 (3.8) | 0.385 |
| Breastfeeding | 1 (0.2) | 0 (0.0) | 0 (0.0) | 0 (0.0) | 1 (1.9) | 0.067 |
| Thrombo-embolic | 1 (0.2) | 1 (0.8) | 0 (0.0) | 0 (0.0) | 0 (0.0) | 0.479 |

*CS, Caesarean Section; LRA, LocoRegional Analgesia; VD, Vaginal Delivery*

^‡^ No woman required additional locoregional analgesia (nerve block), the cases identified were women requiring intravenous opioids.

**Table S.3.** Comparison between the analysed and non-analysed women.

| 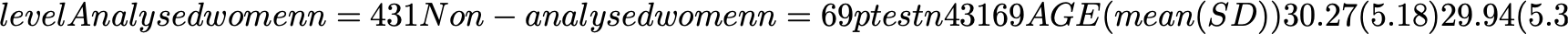 | **Analysed women**  **n = 431** | **Non-analysed women**  **n = 69** | **p** |
| --- | --- | --- | --- |
| Age (years) | 30.3 ± 5.2 | 29.9 ± 5.3 | 0.633 |
| Height (cm) | 164.3 ± 7.1 | 163.1 ± 5.7 | 0.210 |
| Weight (Kg) | 69.5 ± 16.0 | 65.4 ± 18.0 | 0.057 |
| ASA Score |  |  |  |
| ASA 2 | 395 (92.5) | 58 (87.9) | 0.200 |
| ASA 3 | 32 (7.5) | 8 (12.1) |  |
| Cardiovascular history | 3 (0.7) | 0 (0.0) | 1.000 |
| Respiratory history | 20 (4.6) | 3 (4.3) | 1.000 |
| Diabetes | 23 (5.3) | 8 (11.6) | 0.083 |
| Tobacco consumption | 32 (7.4) | 0 (0.0) | 0.038 |
| Tobacco consumption during pregnancy | 20 (4.6) | 0 (0.0) | 0.135 |
| Substance use disorder | 2 (0.5) | 0 (0.0) | 1.000 |
| Number of pregnancies |  |  | 0.889 |
| 1 | 146 (34.1) | 25 (37.9) |  |
| 2 | 122 (28.5) | 19 (28.8) |  |
| 3 | 83 (19.4) | 11 (16.7) |  |
| 4 | 38 (8.9) | 4 (6.1) |  |
| > 4 | 39 (9) | 7 (10.6) |  |
| Foetal malformation | 6 (1.4) | 1 (1.4) | 1.000 |
| Preeclampsia | 9 (2.1) | 1 (1.4) | 1.000 |
| Intrauterine growth restriction | 15 (3.5) | 1 (1.4) | 0.602 |
| Assisted reproductive technology | 11 (2.6) | 3 (4.3) | 0.655 |
| Childbirth education class | 194 (45.0) | 15 (21.7) | 0.001 |
| Labour and delivery modalities |  |  | 0.156 |
| Induced vaginal delivery | 123 (28.7) | 13 (19.7) |  |
| Spontaneous vaginal delivery | 228 (53.3) | 41 (62.1) |  |
| Scheduled CS | 24 (5.6) | 1 (1.5) |  |
| Emergency CS | 53 (12.4) | 11 (16.7) |  |
| Length of labour (hours) | 10.0 ± 6.0 | 14.3 ± 7.5 | <0.001 |
| Operative vaginal delivery | 74 (17.2) | 12 (17.4) | 1.000 |
| Postpartum Haemorrhage | 61 (14.2) | 7 (10.1) | 0.476 |
| Drug Allergy | 1 (0.2) | 0 (0.0) | 1.000 |
| Eclampsia | 2 (0.5) | 0 (0.0) | 1.000 |
| Amniotic embolism | 1 (0.2) | 0 (0.0) | 1.000 |
| Length of stay (days) | 3 [3 – 4] | 4 [4 – 5.5] | 0.064 |

*ASA, American Society of Anesthesiologists; CS, Caesarean Section*

**Table S4.** Inter-item correlations of the ObsQoR-10-French at 24 hours post-delivery.

|  | **Item 1** | **Item 2** | **Item 3** | **Item 4** | **Item 5** | **Item 6** | **Item 7** | **Item 8** | **Item 9** | **Item 10** |
| --- | --- | --- | --- | --- | --- | --- | --- | --- | --- | --- |
| **Item 1** |  | 0.196 | 0.216 | 0.156 | 0.323 | 0.327 | 0.138 | 0.091 | 0.226 | 0.151 |
| **Item 2** | 0.196 |  | 0.464 | 0.481 | 0.240 | 0.222 | 0.228 | 0.166 | 0.227 | 0.160 |
| **Item 3** | 0.216 | 0.464 |  | 0.455 | 0.310 | 0.287 | 0.273 | 0.148 | 0.239 | 0.203 |
| **Item 4** | 0.156 | 0.481 | 0.455 |  | 0.205 | 0.225 | 0.292 | 0.159 | 0.153 | 0.168 |
| **Item 5** | 0.323 | 0.240 | 0.310 | 0.205 |  | 0.427 | 0.406 | 0.351 | 0.313 | 0.489 |
| **Item 6** | 0.327 | 0.222 | 0.287 | 0.225 | 0.427 |  | 0.517 | 0.302 | 0.542 | 0.467 |
| **Item 7** | 0.138 | 0.228 | 0.273 | 0.292 | 0.406 | 0.517 |  | 0.586 | 0.383 | 0.518 |
| **Item 8** | 0.091 | 0.166 | 0.148 | 0.159 | 0.351 | 0.302 | 0.586 |  | 0.236 | 0.553 |
| **Item 9** | 0.226 | 0.227 | 0.239 | 0.153 | 0.313 | 0.542 | 0.383 | 0.236 |  | 0.474 |
| **Item 10** | 0.151 | 0.160 | 0.203 | 0.168 | 0.489 | 0.467 | 0.518 | 0.553 | 0.474 |  |

**Table S5.** Inter-item correlations of the ObsQoR-10-French at 24 hours post-delivery.

|  | **Item 1** | **Item 2** | **Item 3** | **Item 4** | **Item 5** | **Item 6** | **Item 7** | **Item 8** | **Item 9** | **Item 10** |
| --- | --- | --- | --- | --- | --- | --- | --- | --- | --- | --- |
| **Item 1** |  | 0.188 | 0.230 | 0.180 | 0.505 | 0.313 | 0.196 | 0.226 | 0.237 | 0.245 |
| **Item 2** | 0.188 |  | 0.539 | 0.460 | 0.195 | 0.208 | 0.173 | 0.131 | 0.190 | 0.154 |
| **Item 3** | 0.230 | 0.539 |  | 0.450 | 0.266 | 0.331 | 0.305 | 0.249 | 0.126 | 0.206 |
| **Item 4** | 0.180 | 0.460 | 0.450 |  | 0.161 | 0.148 | 0.133 | 0.145 | 0.106 | 0.164 |
| **Item 5** | 0.505 | 0.195 | 0.266 | 0.161 |  | 0.504 | 0.454 | 0.426 | 0.312 | 0.584 |
| **Item 6** | 0.313 | 0.208 | 0.331 | 0.148 | 0.504 |  | 0.672 | 0.468 | 0.492 | 0.520 |
| **Item 7** | 0.196 | 0.173 | 0.305 | 0.133 | 0.454 | 0.672 |  | 0.718 | 0.500 | 0.602 |
| **Item 8** | 0.226 | 0.131 | 0.249 | 0.145 | 0.426 | 0.468 | 0.718 |  | 0.351 | 0.647 |
| **Item 9** | 0.237 | 0.190 | 0.126 | 0.106 | 0.312 | 0.492 | 0.500 | 0.351 |  | 0.428 |
| **Item 10** | 0.245 | 0.154 | 0.206 | 0.164 | 0.584 | 0.520 | 0.602 | 0.647 | 0.428 |  |

**Table S6.** Items responsiveness of the ObsQoR-10-French between prepartum phase and 48 hours post-delivery.

| ObsQoR-10-French items | Before delivery | H24 | Mean Change* | % Change from Baseline | Cohen Effect Size | Standardized Response Mean |
| --- | --- | --- | --- | --- | --- | --- |
| Q1 | 5.8 ± 2.8 | 7.0 ± 2.4 | -1.2 [-1.5 – -0.9] | -20.7 | 0.43 | 0.34 |
| Q2 | 9.1 ± 2.1 | 9.7 ± 1.2 | -0.6 [-0.8 – -0.4] | -6.6 | 0.28 | 0.24 |
| Q3 | 9.7 ± 1.0 | 9.6 ± 1.2 | 0.1 [-0.08 – 0.22] | 1.0 | 0.07 | 0.05 |
| Q4 | 9.2 ± 2.0 | 9.7 ± 1.2 | -0.6 [-0.8 – -0.4] | -5.4 | 0.29 | 0.24 |
| Q5 | 7.2 ± 2.3 | 7.7 ± 2.0 | -0.6 [-0.8 – -0.3] | -6.9 | 0.24 | 0.20 |
| Q6 | 9.2 ± 1.6 | 9.3 ± 1.5 | -0.1 [-0.3 – 0.1] | -1.1 | 0.05 | 0.03 |
| Q7 | 8.6 ± 2.4 | 9.4 ± 1.8 | -0.7 [-1.0 – -0.5] | -9.3 | 0.30 | 0.28 |
| Q8 | 8.7 ± 2.4 | 8.8 ± 2.3 | -0.2 [-0.4 – 0.1] | -1.1 | 0.07 | 0.05 |
| Q9 | 9.7 ± 1.2 | 9.7 ± 1.1 | -0.0 [-0.2 – 0.1] | 0.0 | 0.03 | 0.02 |
| Q10 | 8.4 ± 2.1 | 8.7 ± 1.8 | -0.3 [-0.5 – -0.1] | -3.6 | 0.13 | 0.12 |
| Total | 85.6 ± 10.8 | 89.7 ± 10.5 | -4.2 [-5.5 – -2.9] | -4.8 | 0.39 | 0.32 |

**Confidence interval at 95% between brackets []*

**Figure S1.** Heatmap of inter-item correlations of the ObsQoR-10-French score at 48 hours after delivery. The 10 questions composing the ObsQoR-10-French questionnaire are distributed on the axes. The strength of the correlation between the items is represented by the circle size (larger circle for stronger association) and by the colour shade. Negative correlations are represented in red, and positive correlations in blue.

*
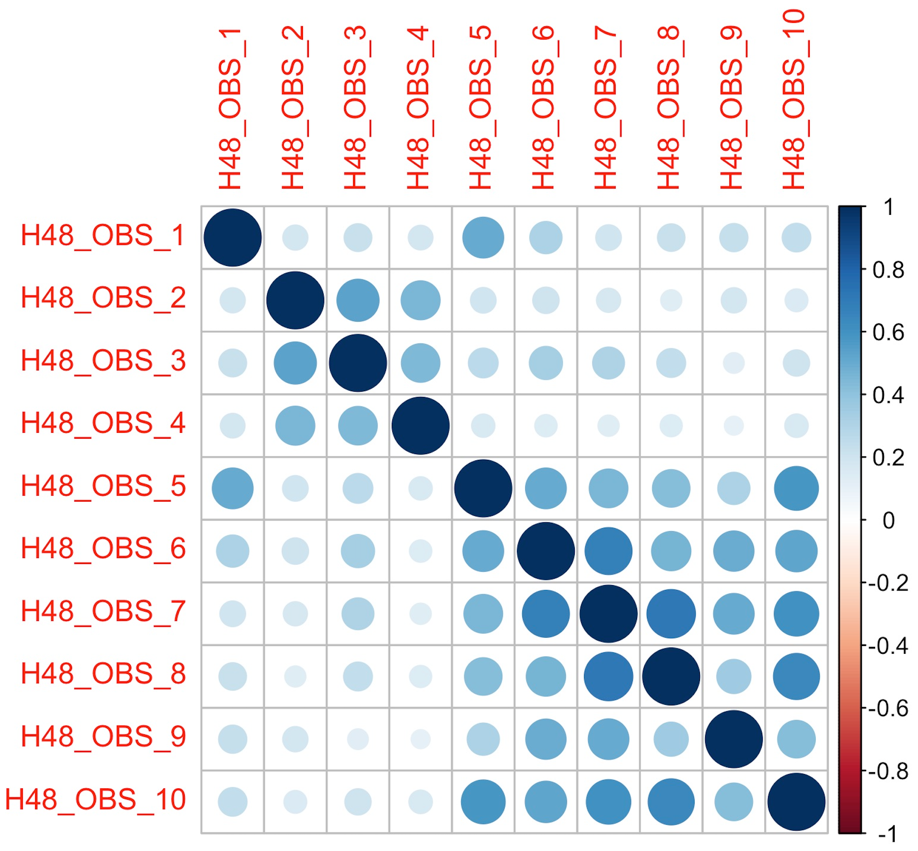
*

**Figure S2.** Screeplot representation of the ObsQoR-10-French at 24 hours after delivery.

*
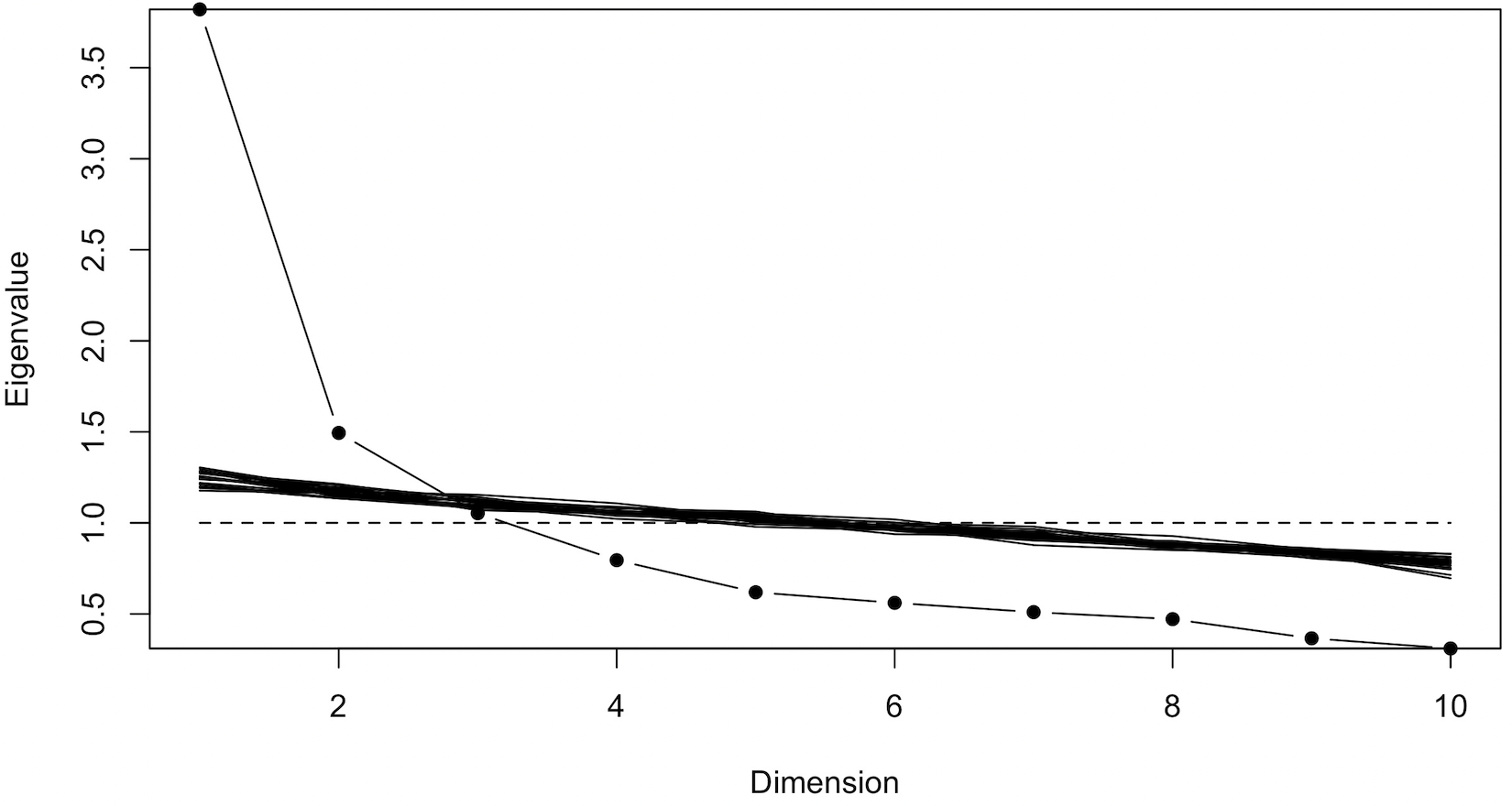
*

**Figure S3.** Screeplot representation of the ObsQoR-10-French at 48 hours after delivery.

*
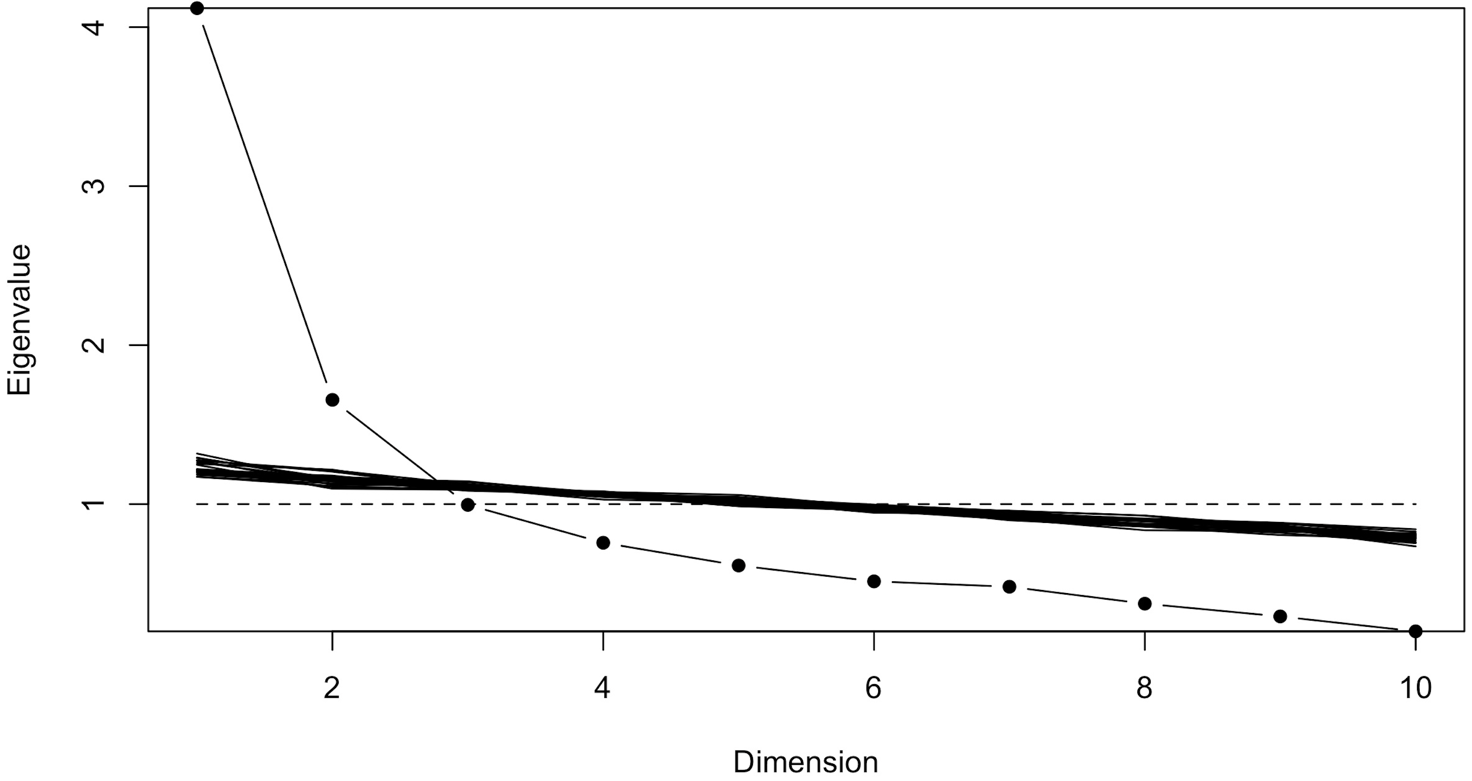
*

**Figure S4.** Distribution of the ObsQoR-10-French score at the three times (prepartum, at 24 hours and at 48 hours post-delivery) depending on the delivery modality: A for spontaneous vaginal delivery, B for induced vaginal delivery, C for elective C-section, D for non-elective C-section.

**Figure S5.** Distribution of the global ObsQoR-10-French at 24 hours post-delivery.


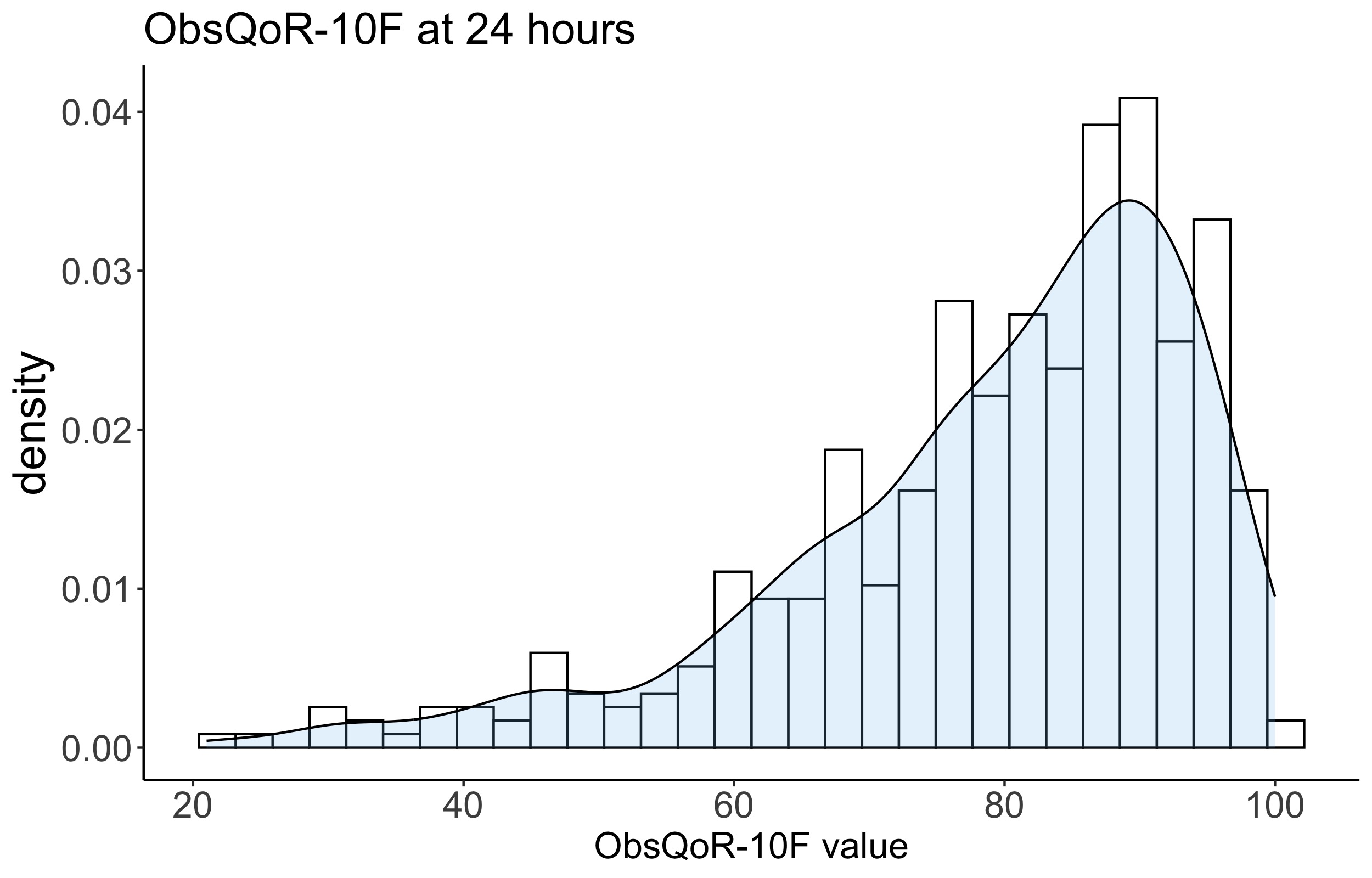


**Figure S6.** Distribution of the global ObsQoR-10-French at 48 hours post-delivery.


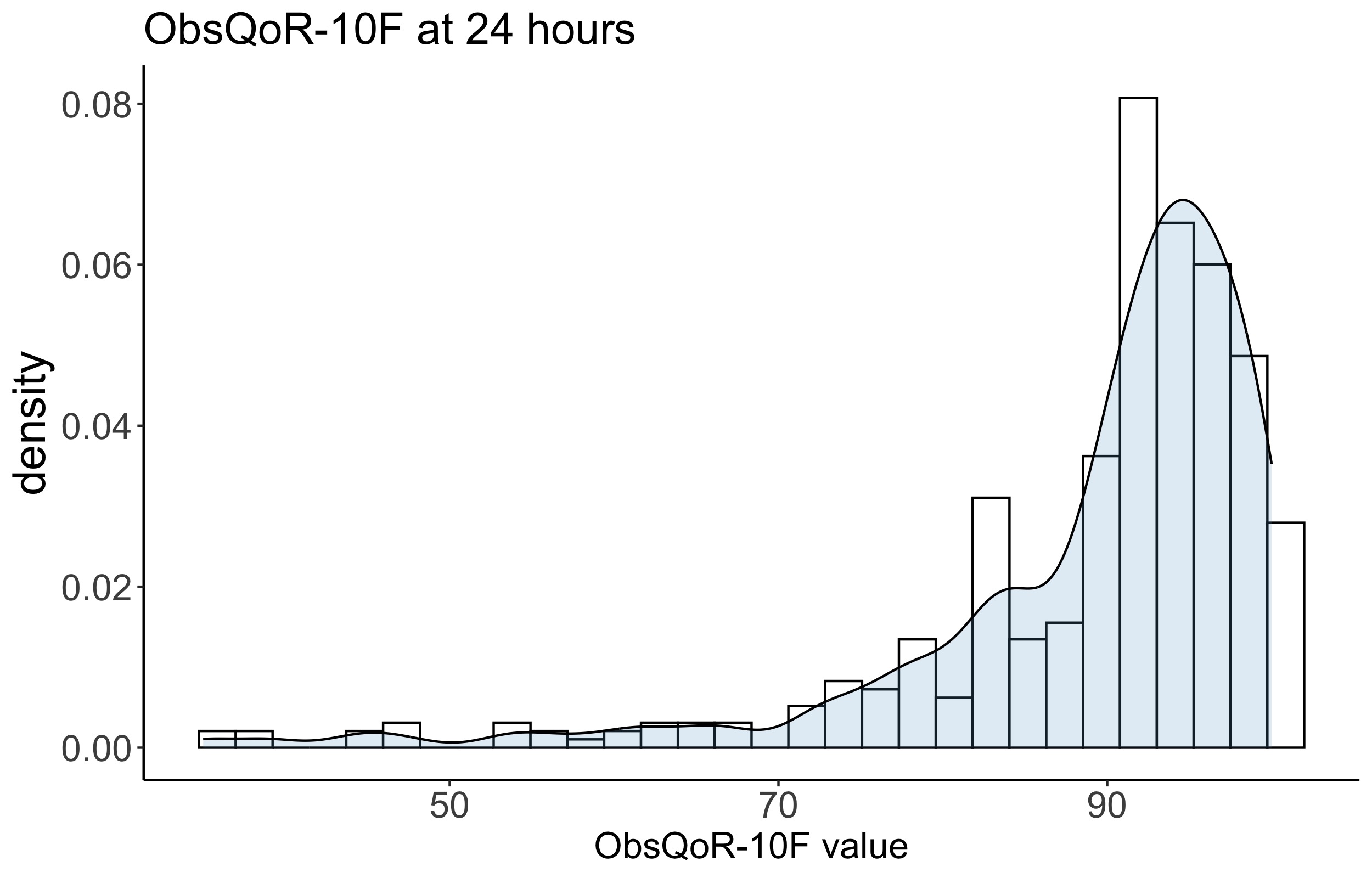

Supplement: Multimedia component 1 [file mmc1.docx]
